# Assessment of data quality in a multi-centre cross-sectional study of participation and quality of life of children with cerebral palsy

**DOI:** 10.1186/1471-2458-6-273

**Published:** 2006-11-06

**Authors:** Heather Dickinson, Kathryn Parkinson, Vicki McManus, Catherine Arnaud, Eva Beckung, Jérôme Fauconnier, Susan I Michelsen, Jackie Parkes, Giorgio Schirripa, Ute Thyen, Allan Colver

**Affiliations:** 1 School of Population and Health Sciences, Newcastle University, 21 Claremont Place, Newcastle, NE2 4AA, UK; 2 School of Clinical Medical Sciences, Newcastle University, 4th Floor William Leech Building, Medical School, Framlington Place, Newcastle, NE2 4HH, UK; 3 Enable Ireland, Lavanagh Centre, Ballintemple, Cork, Ireland; 4 Institut National de la Santé et de la Recherche Medicale, U558 Département de Santé Publique, Faculté de Médecine, 37 allées J Guesde, 31 073 Toulouse, France; 5 Göteborg University, The Queen Silvia Children's Hospital, S-41685 Göteborg, Sweden; 6 Université Joseph Fournier, SIIM-Pole Exploitation, CHU de Grenoble BP 217, 38043 Grenoble cedex 9, France; 7 National Institute of Public Health, Oster Farimagsgade 5, 1399 Copenhagen, Denmark; 8 School of Nursing & Midwifery, Queen's University Belfast, 21 Stranmillis Road, Belfast, BT9 5AF, Northern Ireland; 9 Azienda Sanitaria Locale Viterbo, Viale Trento 18 H, 01100 Viterbo, Italy; 10 Klinik fur Kinder, Universitatsklinikum Schleswig-Holstein, Ratzeburger Allee 160, 23538 Lubeck, Germany; 11 Sir James Spence Institute, Newcastle University, Royal Victoria Infirmary, Newcastle NE1 4LP, UK; 12 (deceased)

## Abstract

**Background:**

SPARCLE is a cross-sectional survey in nine European regions, examining the relationship of the environment of children with cerebral palsy to their participation and quality of life. The objective of this report is to assess data quality, in particular heterogeneity between regions, family and item non-response and potential for bias.

**Methods:**

1,174 children aged 8–12 years were selected from eight population-based registers of children with cerebral palsy; one further centre recruited 75 children from multiple sources. Families were visited by trained researchers who administered psychometric questionnaires. Logistic regression was used to assess factors related to family non-response and self-completion of questionnaires by children.

**Results:**

431/1,174 (37%) families identified from registers did not respond: 146 (12%) were not traced; of the 1,028 traced families, 250 (24%) declined to participate and 35 (3%) were not approached. Families whose disabled children could walk unaided were more likely to decline to participate. 818 children entered the study of which 500 (61%) self-reported their quality of life; children with low IQ, seizures or inability to walk were less likely to self-report. There was substantial heterogeneity between regions in response rates and socio-demographic characteristics of families but not in age or gender of children. Item non-response was 2% for children and ranged from 0.4% to 5% for questionnaires completed by parents.

**Conclusion:**

While the proportion of untraced families was higher than in similar surveys, the refusal rate was comparable. To reduce bias, all analyses should allow for region, walking ability, age and socio-demographic characteristics. The 75 children in the region without a population based register are unlikely to introduce bias.

## Background

SPARCLE [1] is a cross-sectional survey in nine European regions which examines the relationship between the physical, social and attitudinal environment of children with cerebral palsy and their participation in everyday activities and quality of life. Its analyses will take account of family stress and child behaviour. Children with cerebral palsy were studied because they often have impairments of learning, hearing, vision, communication and epilepsy in addition to motor impairment and so are more representative of disabled children than a group with for example just epilepsy or movement difficulties. Importantly, there are also population registers of such children, reducing the risk of selecting a biased study population. Children aged 8–12 years were studied because they have been investigated much less than preschool children, are able to report their own Quality of Life and have not entered adolescence where additional factors operate. The study is original in its methods by directly engaging children themselves, where possible, and including those with communication difficulties and severe learning difficulties even if they cannot self-report.

The aim of this paper is to assess data quality and potential for bias; the specific objectives were to assess (i) heterogeneity between the nine regions, in particular whether inclusion of children from one region who were not identified from a population-based register is likely to have introduced bias, (ii) whether family non-response is likely to have led to a biased sample, (iii) how children who self-complete differ from other children, and (iv) the extent of item non-response.

## Methods

The study methods have been described in detail elsewhere [1] and are summarised briefly below.

### Sample selection

**Figure 1:**
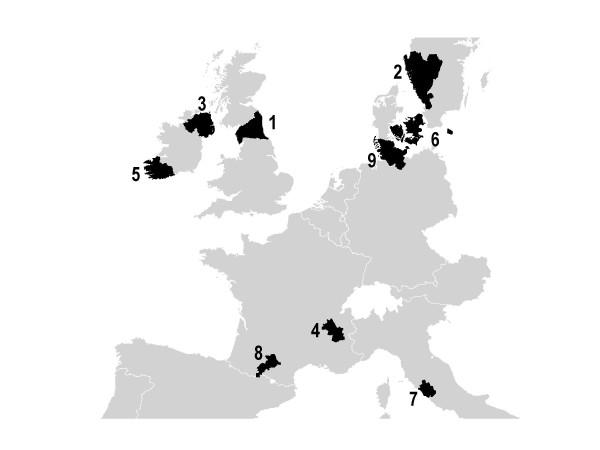
**Map of former European Union (between 1/1/1995 and 1/5/2004) showing regions included in SPARCLE study**. 1 = North England, 2 = Västra Götaland, West Sweden, 3 = Northern Ireland, 4 = Isere, South East France, 5 = Cork and Kerry, South West Ireland, 6 = Sealand, Fynen and Islands, East Denmark, 7 = Central Italy, 8 = Haute Garonne, South West France, 9 = Schleswig-Holstein, North West Germany. File is called: Colver Figure Map.ppt

Participants were selected from eight registers of children with cerebral palsy resident in defined regions: North England, West Sweden, Northern Ireland, South East France, South West Ireland, East Denmark, Central Italy, South West France (see Figure 1 : Map of former European Union (between 1/1/1995 and 1/5/2004) showing regions included in SPARCLE study) [2]. A further region in North West Germany joined the study but its sample could not be drawn from a population-based register and was constructed from referrals from multiple sources.

Milder cerebral palsy is more common so, in regions with sufficient registered cases (North England and East Denmark), similar numbers of children were sampled at each level of severity: children were grouped by walking ability and a random sample selected from each stratum as shown in Table 1. West Sweden and Northern Ireland selected a random sample of children in the highest strata of walking ability and approached all children in the remaining strata. In regions with fewer registered cases, all children were approached.

Children with dates of birth between 31/07/1991 and 01/04/97 inclusive were eligible. Families were interviewed between May 2004 and August 2005 when, if possible, the child was over eight and under thirteen years. The children initially selected comprised the *initial sample*. As several attempts were made to contact eventual non-responders, often over a long period of time, they were assigned a notional interview date of 1st January 2005. Children were excluded if they were born outside the specified dates of birth and they were also over six months outside the specified age range on the interview date, or if they had died, been misdiagnosed, moved out of the area, or their parents had language difficulties. The remaining children comprised the *final sample*.

### Interview of children and parents

Researchers were trained together before visiting the selected families at home to administer questionnaires to parents and, if possible, children. Background information on the socio-demographic characteristics of the families and the impairment of the children was recorded. Children's ability to report their own Quality of life was assessed [3,4]. If possible, their self-reported Quality of life was captured using the KIDSCREEN questionnaire [5], a modern instrument with excellent psychometric properties assessing children's quality of life on ten dimensions, whose questions were derived from focus group work with children across Europe. Two additional questions, similar to questions in the Child Health Questionnaire[6], captured children's experience of pain. Parental reports of children's Quality of life, participation, frequency of participation in discretionary activities, environment, general health and behaviour were recorded using the KIDSCREEN[5], Life-H[7], Frequency of Participation [1], European Child Environment [8-10], Child Health[6], and Strengths and Difficulties [11] questionnaires respectively. Additionally, parent reports of their children's pain were recorded if the children were unable to report their own pain. Parental stress was recorded using the Parental Stress Index – Short Form questionnaire [12]. Participation and Quality of life were outcomes; environment was the main exposure; impairment, pain, socio-demographic characteristics, frequency of participation in discretionary activities, the child's behaviour, and parental stress were regarded as potential effect modifiers.

### Statistical Methods

#### Heterogeneity between regions: socio-demographic characteristics and family non-response

We assessed heterogeneity of socio-demographic characteristics and non-response between regions. For all regions, we considered: parental educational qualifications; employment; area of domicile; type of school attended by child (these were dichotomised respectively as minimal school-leaving qualification or lower/other; at least one parent working full-time or two working part-time/other; urban/rural; mainstream/special unit in mainstream school or special school). We also considered rates of completion of questionnaires by children. For all regions except North West Germany, where children were not selected from a population-based register, we considered non-response, both overall and due to: (i) failure to trace the family (*non-traceability*), (ii) traced families declining to participate (*refusal*), and (iii) traced families not being approached by researchers (*non-approach*). The significance of heterogeneity between regions was assessed using chi-squared tests. The regions contributing to any significant heterogeneity were identified using a comparative risk function (CRF) [13], which compares the odds of children in a specific region being in a specific category with the corresponding odds for the entire sample. This method has the advantage of comparing each region with the entire sample and yielding confidence intervals which are independent, whereas logistic regression compares each region with one which is arbitrarily selected as the reference category and yields confidence intervals which are correlated.

#### Predictors of family non-response

We used logistic regression to assess whether non-traceability and refusal were related to age, gender and *level of impairment *(walking ability, presence of seizures, impairment of vision and hearing) recorded by the register at age 4–5 years. We assessed whether any such associations could explain the differences in response rates between regions [14]; odds ratios (ORs) and 95% confidence intervals (CI) are reported.

For each region, the method of approach to families was classified as direct (initial contact with the family was by the researcher) or indirect (initial contact was by a doctor, social worker or therapist) – see Table 2. Logistic regression was then used to assess whether non-traceability or refusal was related to the method of approach.

We calculated weights which allowed for (i) the sampling strategy and (ii) both the sampling strategy and non-response. Use of weights usually reduces bias but increases the variance of estimates. We estimated the probable loss of efficiency (percentage increase in variance) which would be induced by using weights when they are not necessary to reduce bias [15].

#### Characteristics of children not recorded by registers and ineligible children

We assigned weights which allowed for the sampling strategy to children selected from population-based registers, so that the weighted observations represented the characteristics of children on the registers who would have been included in the study in the absence of sampling. We then compared the age, gender and level of impairment as assessed at interview, of children in North West Germany with that of children in the weighted sample [15]. Logistic regression was also used to assess whether the few included children who did not meet the inclusion criteria differed from eligible respondents.

#### Characteristics of self-completing children

Children with learning difficulties were expected to have difficulty in completing the KIDSCREEN questionnaire themselves. We therefore used univariate logistic regression to examine how the proportion of children completing the KIDSCREEN questionnaire varied with the children's IQ. We then assessed whether self-completion depended on age, gender and level of impairment as assessed at interview, after allowing for IQ. Finally, we assessed whether the various aspects of impairment were independent predictors of self-completion by removing each one in turn from a multivariable logistic regression and referring to the likelihood ratio test statistic.

#### Item non-response and missing scores

For parents and children who responded, we calculated the proportion of items not completed on each questionnaire. Many of the questionnaires are scored using algorithms which allow calculation of domain scores even if responses to a few items are missing. Therefore we also calculated the proportion of missing scores for each questionnaire.

To mitigate the effects of multiple analyses, a p-value < 0.01 was regarded as significant in all analyses. Statistical analyses were performed using Stata 9 [16].

#### Consent and Ethics approval

The research was carried out in compliance with the Helsinki Declaration. All parents gave written consent. All children with sufficient cognitive capacity gave written consent or communicated consent if unable to write. Ethics committee approvals were obtained in each country.

## Results

The study population, initial sample selected, reasons for exclusion after sampling and final sample are summarised in Table 1. The final sample comprised 1,249 children: 1,174 children selected from the registers, plus 75 children recruited by North West Germany. Due to administrative error, 27 of these children (24 responders and 3 non-responders) were born outside the specified time period and 40 children were interviewed when their age was up to six months outside the specified age range.

### Non-response of families selected from population-based registers

Of the 1,174 included families, 431 (37%) did not respond: 146 (12%) could not be traced; of the 1,028 traced families, 250 (24%) declined to participate and 35 (3%) were not approached by researchers – 11 because professionals requested this, 6 because foster parents cared for them, 6 because the children reached 13 years before the family was approached, and 12 for other reasons. There was significant heterogeneity between regions in rates of non-response, non-traceability and refusal (p < 0.001). Table 3, section (a), shows that compared to all included families, West Sweden and Northern Ireland had a lower response, largely because families who were approached often declined to participate, whereas South West Ireland, East Denmark and Central Italy had a higher response because of a combination of effective tracing of families and a high level of co-operation among those who were traced. Decisions not to approach traced families also appeared to be influenced by regional factors: 10 of the 11 families which professionals requested should not be approached were in one region and 5 of the 6 children in foster care were in another region.

Rates of tracing families did not vary significantly (p > 0.01) with any of the variables considered (see Table 4). Among traced families, those whose children walked using aids or could not walk were significantly less likely to refuse; (OR for refusal compared to children who walked without aids = 0.5, 95%CI: 0.4 to 0.8 and 0.6, 95%CI: 0.4 to 0.9 respectively). The gender, epileptic status, vision and hearing of the disabled child were not significantly associated with refusal. The significant heterogeneity between regions in rates of refusal remained after adjusting for walking ability (p < 0.001).

The five regions which used a direct approach had similar rates of non-traceability to the three regions which used an indirect approach (average rates of 13% and 12% respectively), both unadjusted (see Table 4) and after adjusting for age. Likewise, the direct and indirect approaches resulted in similar rates of refusal (24% and 25% respectively), both unadjusted and after adjusting for age and walking ability. As recent events in Sweden (discussed below) [17] may have led to the high refusal rate (52%) there, we repeated these comparisons excluding Sweden and found a lower refusal rate (12%) in the four regions which used a direct approach (OR for refusal comparing indirect with direct approaches = 2.4, 95%CI: 1.7 to 3.5). Findings were similar when overall non-response of traced families (both refusal and non-approach) was considered.

The estimated percentage increases in variance induced by using sampling weights to allow for the sampling strategy and, additionally, non-response, were 22% and 32% respectively.

### Socio-demographic characteristics of all included children and families

Among the 818 responders in the final sample, the mean age of the children on date of interview was 10 1/2 years; 59% were boys and 41% girls. The distribution of age and gender was similar across regions. Table 3, section (b), shows there was heterogeneity between regions in terms of educational qualifications and employment status of parents, whether the environment was predominantly urban or rural and type of school attended by the child. Compared to all included families, parents in North England and Northern Ireland tended to have poorer educational qualifications and to be less likely to work full-time, whereas those in East Denmark and West Sweden tended to have better educational qualifications and those in South West France were more likely to work full-time. South East France and South West Ireland had lower proportions of families living in urban environments, whereas North England had a higher proportion. South West Ireland and Central Italy had lower proportions of children in special units in mainstream schools or in special schools, whereas Northern Ireland, South East France and North West Germany had higher proportions.

### Characteristics of children not identified from registers and ineligible children

Weighted logistic regression indicated that age, gender and levels of impairment, as assessed at interview, among children recruited in North West Germany were similar to those of eligible children selected from the population-based registers.

24 (3%) of the children of included responders were not strictly eligible as they were born outside the specified time period. Logistic regression, comparing these ineligible children with eligible children, showed no indication that they differed in terms of impairment, as assessed at interview, or gender.

### Completion by children

500/818 (61%) children completed the KIDSCREEN questionnaire, with some variation in self-completion rates between regions as shown in Table 3, section (c). Children with IQ less than 70 were much less likely than other children to complete the questionnaires. Completion rates did not vary significantly with the children's age or gender. Children who were unable to walk and those with seizures, impaired hearing or vision were less likely to complete the questionnaires (see Table 4); but only inability to walk, seizures and vision remained significant after allowing for IQ and only IQ, inability to walk and seizures were independently significant (p < 0.01).

### Item non-response

Age and gender were reported for all 818 children included in the final sample. Only eight children (0.4%) had missing data on impairment items. For questionnaires administered to parents, the proportion of items not completed ranged from 0.4% for the impairment questionnaire to 5% for the KIDSCREEN questionnaire. Children failed to complete 2% of items (see Table 5).

### Missing scores

The proportion of missing scores on questionnaires completed by parents and children varied from 2% (for Life-H, Strengths and Difficulties Questionnaire, Parental Stress Index) to 7% for KIDSCREEN administered to parents. Questions on the KIDSCREEN questionnaire about finance had the poorest response: scores were missing for 11% and 21% of children and parents respectively. Questions on Life-H about community life also had a relatively poor response, with 20% of scores missing.

## Discussion

### Family non-response

The overall rate of non-response among families was 37%: 12% non-traceability and 24% refusal and 3% non-approach among those traced. These non-response rates should be evaluated by comparison with surveys of a similar design, which target specific families and conduct face-to-face interviews. A recent British national survey of the mental health of over 12,000 children sampled specific families and conducted face-to-face interviews with parents and children [18]. This survey reported that 5% of families could not be traced and 28% of those traced either opted-out or refused an interview or were not approached. Hence its refusal rate for families was similar to that of SPARCLE, but it traced a higher proportion of families, probably because its sample was drawn from child benefit records, so the families had an incentive to keep their address records up-to-date.

The identification of cases from population-based registers which ascertain cases from multiple sources mitigates against selection bias but cannot reduce non-response bias. Although we wished to compare the level of non-response in our study with that in similar epidemiological studies which identified children from population-based registers and administered face-to-face interviews, we were unable to find reports of any such studies. Hence we are unable to comment on whether our findings are typical of such register-based studies.

Many national surveys of households conducted in the 1990s reported lower non-contact rates (typically 2% to 8%) and lower refusal rates (typically 2% to 29%) than SPARCLE [19,20]. These surveys usually attempted to contact residents at specific addresses whereas SPARCLE attempted to trace specific families. Further, they were often conducted by telephone or mail and some allowed proxy respondents within households. All these factors would probably have led to higher response rates.

Families whose children could walk without aids were less likely to agree to participate. Refusal is influenced by individual, household and societal factors: men, people with lower educational qualifications, those from ethnic minorities and those living in urban areas are often less likely to co-operate [19,20]. We were unable to record these factors for non-respondents in SPARCLE.

Rates of non-traceability and refusal varied significantly between regions. This is not surprising as the method of approach varied substantially between regions, reflecting variation not only in national legislation interpreting the European directive on data privacy [21,22], but also in implementation of legislation in different regions. Overall, rates of non-traceability and refusal were similar regions which approached families directly and indirectly. However, the level of refusal in Sweden, where families were approached directly, was twice that in other regions; this was almost certainly due to recent controversial events when a judicial judgement demanded release of patient records [17]. Excluding Sweden, regions which approached families directly had about half the refusal rate of other regions. This finding should be interpreted cautiously, firstly because it follows exclusion of a region after noting its unusual data and, secondly, because it is based on comparison of only seven remaining regions.

### Possible bias

Results of analyses could be subject to bias if the sampling strategy is not taken into account, or if non-response or inclusion of unrepresentative children resulted in a biased sample.

It is possible to adjust for the sampling strategy either by using sampling weights or by adjusting for the design variables (region and walking ability) [15]. If it is assumed that non-response depends only on observed variables – and we have shown that it is related to region, age and walking ability – similar strategies can be used to allow for the effects of non-response. Sampling weights generally reduce bias but increase the variance of estimates. Allowing for the sampling strategy and non-response by using weights would probably increase the variance of estimates by about a third; therefore adjustment for region, walking ability and age may be preferred.

The age, gender and levels of impairment of children in North West Germany, who were identified from referrals from multiple sources, were similar to those of children recorded on population-based registers. Children who were ineligible because their date of birth was outside the specified range were similar to other included children in terms of gender and impairment. Therefore it is unlikely that inclusion of these children has introduced any substantial bias.

The proportion of missing items and scores among respondents was low. The correlates and possible effects of these missing data will be evaluated in reports considering each questionnaire.

### Heterogeneity between regions

The socio-demographic characteristics of the families varied substantially between regions. Census data show that, in North East England and Northern Ireland, a high proportion of families with dependent children have no working adults [23] and a high proportion of adults have no educational qualifications [24,25]. Therefore it is not surprising that the SPARCLE samples from North England (where the majority of the population live in the North East part) and Northern Ireland compared unfavourably with other European regions in these respects. The high proportion of families with at least one parent working in South West France is probably due to a combination of factors: this region had the highest proportion of included children with good walking ability (47%) and a high proportion of mothers with good educational qualifications who are more likely to work; it is also very well equipped in terms of institutional care for handicapped children [26]. The higher proportion of parents in Denmark and Sweden who had qualifications above the minimum probably reflects the high level of participation in job-related continuing education in those countries [27]. The type of school attended by the child also varied substantially between regions, partly reflecting variation in national policies [28].

This heterogeneity between regions is a strength of the study, giving greater statistical power to examine the effects of factors which, if improved, would yield the greatest benefit to disabled children and their families. It was not the purpose of the SPARCLE study to draw conclusions about differences between regions or countries and, indeed, it would be misleading to do so, because some of the apparent differences reported in Table 3 may be chance findings consequent to multiple analyses and some may be due to the effects of differences between regions in socio-demographic characteristics. As other differences between regions, not recorded by the study, are highly likely, it is important to adjust for region in all analyses.

### Children's self-report

Although we attempted to use objective criteria [3,4] to indicate whether a child could self-report, interviewers reported that some children who satisfied the criteria did not always understand the questions and their self reports were therefore excluded. Nevertheless, self-completion rates were similar between regions. As expected, children with lower IQ were less likely to self-complete. However, somewhat surprisingly, after allowing for IQ, children who were unable to walk and those with seizures were less likely to self-complete.

### Comparison with other studies

While a few regional and national studies evaluate whether non-response is likely to have introduced bias [24,29], and some studies report the extent of non-response without evaluation of its effects [18,30-32], most small studies of disabled children which we identified did neither.

We did not find any evaluations of differences in response rates between surveys which used direct and indirect approaches to potential participants. As ethical requirements concerning recruitment of participants in clinical studies are now influenced by recent European directives on data privacy [21] and good clinical practice [33], surveys may be more frequently required to approach potential participants indirectly. The consequent effect on response rates is of considerable interest.

## Conclusion

While refusal rates were comparable to those in similar surveys, non-traceability rates were higher, probably because SPARCLE identified potential participants from population-based case registers which may not have had up-to-date addresses of those who moved after registration. Item non-response was low. The small number of ineligible children and children not selected from population-based registers are unlikely to introduce bias.

To reduce bias due to the sampling strategy, differential non-response, and heterogeneity between regions, all analyses should allow for region as either a fixed or random effect, allow for walking ability and age either by adjusting or by using sampling weights, and evaluate whether further adjustment for socio-demographic characteristics is advisable.

Research is needed to evaluate whether indirect approaches to families result in lower response rates than direct approaches. Studies should routinely report their method of approach to participants and their response rates, to allow meta-analysis of response rates sub-grouped by type of approach.

Surveys rarely assess data quality prior to analysis and they should routinely do so.

## Competing interests

The author(s) declare that they have no competing interests.

## Authors' contributions

HD managed the data performed all statistical analyses and wrote the paper. KP managed the data, managed the acquisition of data in her region and revised the manuscript for important intellectual content. VM managed the acquisition of data in her region and collated methods of approaching families in each centre. CA managed the acquisition of data in her region and revised the manuscript for important intellectual content. EB managed the acquisition of data in her region. JF managed the acquisition of data in his region. SM managed the acquisition of data in her region. JP managed the acquisition of data in her region. GS managed the acquisition of data in his region. UT managed the acquisition of data in her region. AC conceived the study, participated in its design, directed the project and revised the manuscript for important intellectual content. All authors read and approved the final version.

## Pre-publication history

The pre-publication history for this paper can be accessed here:



## Figures and Tables

**Table 1:** Selection and inclusion of participants, by region

	**Population-based registers**																					
					
	**North England**		**West Sweden**		**Northern Ireland**		**South East France**		**South West**		**East Denmark**		**Central Italy**		**South West France**		**All registers**		**North West Germany**		**ALL**	
	**No.**	*(%)*	**No.**	*(%)*	**No.**	*(%)*	**No.**	*(%)*	**No.**	*(%)*	**No.**	*(%)*	**No.**	*(%)*	**No.**	*(%)*			**No.**	*(%)*	**No.**	*(%)*

**Study population**	**437**		**290**		**284**		**125**		**127**		**396**		**108**		**117**		**1,884**		**75**		**1,959**	
Walking ability as recorded by the registry:																						
Walks unaided	254		198		187		63		72		246		43		65		1,127					
Walks using aids	86		46		45		19		18		57		28		34		333		-		-	
Unable to walk	97		46		52		43		37		92		35		18		420		-		-	
Unknown	-		-		-		-		-		-		2		-		2		-		-	
Initial sample*	**186**	*(43)*	**220**	*(76)*	**203**	*(71)*	**125**	*(100)*	**127**	*(100)*	**165**	*(42)*	**108**	*(100)*	**117**	*(100)*	**1,251**		**75**	*-*	**1,326**	
Walking ability as recorded by the registry:																						
Walks unaided	87		128		106		63		72		80		43		65		644					
Walks using aids	50		46		45		19		18		38		28		34		278		-		-	
Unable to walk	49		46		52		43		37		47		35		18		327		-		-	
Unknown	-		-		-		-		-		-		2		-		2		-		-	
Excluded after sampling**	**8**	*(4)*	**4**	*(2)*	**4**	*(2)*	**4**	*(3)*	**19**	*(15)*	**22**	*(13)*	**10**	*(9)*	**6**	*(5)*	**77**	*(6)*	-		**77**	*(6)*
Dead	0		2		1		1		3		10		3		1		21		-		21	
Misdiagnosed	4		1		3		3		4		2		2		0		19		-		19	
Moved out of area	4		1		0		0		5		7		4		4		25		-		25	
Parental language difficulty	0		0		0		0		0		3		0		1		4		-		4	
Other	0		0		0		0		7		0		1		0		8		-		8	
Final sample**	**178**	*(96)*	**216**	*(98)*	**199**	*(98)*	**121**	*(97)*	**108**	*(85)*	**143**	*(87)*	**98**	*(91)*	**111**	*(95)*	**1,174**	*(94)*	**75**	*(100)*	**1,249**	*(94)*
Responders^†^	**116**	*(65)*	**83**	*(38)*	**102**	*(51)*	**67**	*(55)*	**98**	*(91)*	**115**	*(80)*	**85**	*(87)*	**77**	*(69)*	**743**	*(63)*	**75**	*(100)*	**818**	*(65)*
Non-responders^†^	**62**	*(35)*	**133**	*(62)*	**97**	*(49)*	**54**	*(45)*	**10**	*(9)*	**28**	*(20)*	**13**	*(13)*	**34**	*(31)*	**431**	*(37)*	**-**		**431**	*(35)*

Percentages are relative to number in: * study population; ** initial sample; † final sample.

a One child was excluded as the walking ability was not recorded.

b Born outwith specified dates of birth and over 6 months outside specified age range on interview date.

**Table 2:** Method of initial approach to families, by region

**Region**	**Method of approach**		
**North England**	Indirect	Telephone	Child's paediatrician phoned; followed by phone call from researcher
**West Sweden**	Direct	Letter – opt-out	Researcher sent opt-out letter, followed by phone call
**Northern Ireland**	Indirect	Letter – opt-in	Child's primary notifier to the register sent opt-in letter, follow-up letters and in some cases telephoned some families
**South East France**	Direct	Letter – opt-out	Researcher sent opt-out letter, followed by phone call
**South West Ireland**	Direct	Telephone	Researcher phoned, followed by letter
**East Denmark**	Direct	Letter – opt-out	Researcher sent opt-out letter, followed by phone call
**Central Italy**	Indirect	Telephone	Doctor, social worker or therapist phoned family, followed by phone call by researcher
**South West France**	Direct	Telephone	Researcher phoned, followed by letter
**North West Germany**	Indirect	Face-to-face	Child's primary carer approached family face-to-face

**Table 3:** Comparative risk functions comparing non-response, demographic characteristics and self-completion by region with entire study population

	**(a) Non-response**											
				
	**Overall non-response**			**Non-response due to failure to trace families**			**Non-response of traced families**					
	**%**	**CRF**	**95%CI**	**%**	**CRF**	**95%CI**	**%**	**CRF**	**95%CI**			

**North England**	35	0.9	(0.7 to 1.2)	***22***	***2.0***	***(1.5 to 2.8)***	**15**	**0.6**	**(0.4 to 0.9)**			
**West Sweden**	***62***	***2.8***	***(2.2 to 3.5)***	***16***	***1.4***	***(1.0 to 1.9)***	***52***	***3.4***	***(2.6 to 4.3)***			
**Northern Ireland**	***49***	***1.6***	***(1.3 to 2.1)***	**5**	**0.3**	**(0.2 to 0.6)**	***41***	***2.2***	***(1.7 to 2.8)***			
**South East France**	45	1.4	(1.0 to 1.9)	***19***	***1.7***	***(1.1 to 2.5)***	21	0.8	(0.5 to 1.3)			
**South West Ireland**	**9**	**0.2**	**(0.1 to 0.3)**	**2**	**0.1**	**(0.0 to 0.5)**	**6**	**0.2**	**(0.1 to 0.4)**			
**East Denmark**	**20**	**0.4**	**(0.3 to 0.6)**	8	0.6	(0.4 to 1.1)	**8**	**0.3**	**(0.1 to 0.5)**			
**Central Italy**	**13**	**0.3**	**(0.1 to 0.5)**	8	0.6	(0.3 to 1.3)	**6**	**0.2**	**(0.1 to 0.4)**			
**South West France**	31	0.8	(0.5 to 1.1)	15	1.3	(0.8 to 2.1)	16	0.6	(0.3 to 1.0)			
**North West Germany**												
**All centres**	37	1.0	-	12	1.0	-	24	1.0	-			
*p for heterogeneity*			<0.001			<0.001			<0.001			

	**(b) Socio-demographic characteristics**											
	
	**Parental educational qualifications above the minimum**			**At least one parent working full-time (or equivalent)**			**Family living in urban environment**			**Child goes to special school or special unit in mainstream school**		

	**%**	**CRF**	**95%CI**	**%**	**CRF**	**95%CI**	**%**	**CRF**	**95%CI**	**%**	**CRF**	**95%CI**

**North England**	**61**	**0.5**	**(0.4 to 0.7)**	**57**	**0.4**	**(0.3 to 0.5)**	***88***	***3.9***	***(2.3 to 6.8)***	41	0.8	(0.6 to 1.1)
**West Sweden**	***91***	***3.3***	***(1.5 to 7.0)***	78	1.1	(0.7 to 1.8)	70	1.2	(0.8 to 1.9)	52	1.2	(0.8 to 1.8)
**Northern Ireland**	**51**	**0.3**	**(0.2 to 0.5)**	**66**	**0.6**	**(0.4 to 0.8)**	60	0.8	(0.6 to 1.2)	***58***	***1.5***	***(1.1 to 2.2)***
**South East France**	73	0.9	(0.5 to 1.4)	84	1.5	(0.8 to 2.8)	**52**	**0.6**	**(0.4 to 0.9)**	***67***	***2.3***	***(1.4 to 3.7)***
**South West Ireland**	71	0.8	(0.5 to 1.1)	78	1.1	(0.7 to 1.7)	**56**	**0.7**	**(0.5 to 1.0)**	**28**	**0.4**	**(0.3 to 0.7)**
**East Denmark**	***96***	***6.9***	***(2.8 to 17)***	84	1.6	(1.0 to 2.5)	67	1.1	(0.8 to 1.6)	54	1.3	(0.9 to 1.9)
**Central Italy**	80	1.2	(0.8 to 2.1)	81	1.3	(0.7 to 2.1)	66	1.0	(0.7 to 1.6)	**7**	**0.1**	**(0.0 to 0.2)**
**South West France**	84	1.6	(0.9 to 3.0)	***92***	***3.5***	***(1.5 to 7.9)***	61	0.8	(0.5 to 1.3)	45	0.9	(0.6 to 1.4)
**North West Germany**	84	1.6	(0.9 to 3.0)	85	1.7	(0.9 to 3.2)	55	0.7	(0.4 to 1.0)	***80***	***4.5***	***(2.6 to 7.8)***
**All centres**	76	1.0	-	77	1.0	-	65	1.0	-	47	1.0	-
*p for heterogeneity*			<0.001			<0.001			<0.001			<0.001

	**(c) Completion of KIDSCREEN by child**											
										
	**%**	**CRF**	**95%CI**									

**North England**	67	1.3	(0.9 to 1.9)									
**West Sweden**	55	0.8	(0.5 to 1.2)									
**Northern Ireland**	57	0.8	(0.6 to 1.2)									
**South East France**	57	0.8	(0.5 to 1.3)									
**South West Ireland**	68	1.4	(0.9 to 2.1)									
**East Denmark**	***70***	***1.5***	***(1.0 to 2.2)***									
**Central Italy**	**47**	**0.6**	**(0.4 to 0.8)**									
**South West France**	61	1.0	(0.6 to 1.5)									
**North West Germany**	60	1.0	(0.6 to 1.5)									
**All centres**	61	1.0	-									
*p for heterogeneity*			0.02									

CRF = Comparative risk function

CRF <1 and >1 indicate proportions below and above the mean respectively; proportions significantly below the mean are show n in **bold; **proportions significantly above the mean are show n in ***bold and italics***.

**Table 4:** Rates and univariate odds ratios for non-traceability and refusal and for self-completion of Kidscreen questionnaire

	**Non-traceability **^**a**^			**Refusal **^**a**^			**Child completion of KIDSCREEN**		
	**%**	**OR**	**(95% CI)**	**%**	**OR**	**(95% CI)**	**%**	**OR**	**(95% CI)**

***Child characteristics***									
Gender									
Male	13	1.0	-	23	1.0	-	59	1.0	-
Female	12	0.9	(0.6 to 1.3)	26	1.2	(0.9 to 1.5)	64	1.2	(0.9 to 1.6)
Age in years									
7 and 8	7	1.0	-	27	1.0	-	57	1.0	-
9	13	1.9	(1.0 to 3.5)	19	0.6	(0.4 to 1.0)	66	1.5	(1.0 to 2.3)
10	12	1.7	(0.9 to 3.1)	19	0.6	(0.4 to 1.0)	56	1.0	(0.6 to 1.5)
11	13	1.9	(1.0 to 3.4)	26	1.0	(0.6 to 1.5)	66	1.5	(0.9 to 2.3)
12 and 13	17	2.5	(1.4 to 4.5)	30	1.2	(0.8 to 1.8)	61	1.2	(0.8 to 1.8)
Walking ability ^b^									
Walks unaided	14	1.0	-	29	1.0	-	80	1.0	-
Walks using aids	11	0.8	(0.5 to 1.3)	18	0.5	(0.4 to 0.8)	67	0.5	(0.3 to 0.8)
Unable to walk	11	0.8	(0.5 to 1.2)	21	0.6	(0.4 to 0.9)	28	0.09	(0.07 to 0.14)
Seizures ^b^									
No	12	1.0	-	25	1.0	-	74	1.0	-
Yes	12	1.1	(0.7 to 1.6)	25	1.0	(0.7 to 1.4)	29	0.1	(0.1 to 0.2)
Vision problems ^b^									
No	12	1.0	-	24	1.0	-	65	1.0	-
Yes	11	0.8	(0.5 to 1.5)	27	1.2	(0.8 to 1.8)	7	0.03	(0.01 to 0.1)
Hearing problems ^b^									
No	13	1.0	-	27	1.0	-	62	1.0	-
Yes	9	0.7	(0.2 to 2.2)	19	0.6	(0.3 to 1.6)	22	0.2	(0.06 to 0.5)
IQ									
>70	-	-	-	-	-	-	94	1.0	
50 to 70	-	-	-	-	-	-	65	0.1	(0.1 to 0.2)
<50	-	-	-	-	-	-	5	0.003	(0.002 to 0.007)
***Centre characteristics***									
Method of approach									
Direct	13	1.0	-	24	1.0		-	-	-
Indirect	12	0.9	(0.7 to 1.3)	25	1.1	(0.8 to 1.4)	-	-	-

a Excluding North West Germany, where children were not sampled from a population-based register

b For assessment of response, impairment at age 4–5 years was obtained from the register; for assessment of child completion, impairment was obtained from interview.

**Table 5:** Percentage of items not completed and scores missing in each questionnaire

	**Parent**		**Child**	
**Questionnaire**	**% items not completed**	**% scores missing**	**% items not completed**	**% scores missing**

Background	2	*		
Impairment	0.4	*		
Life-H	1	2		
Frequency of Participation Questionnaire	1	*		
Strengths and Difficulties Questionnaire	2	2		
Parental Stress Index (Short Form)	1	2		
European Child Environment Questionnaire	3	*		
KIDSCREEN	5	7	2	2
Pain	3	*	2	*
Child Health Questionnaire	3	3		

* Not scored
